# Supplementary material for: IL-17A-producing NKp44(−) group 3 innate lymphoid cells accumulate in Familial Adenomatous Polyposis duodenal tissue
Source: Nat Commun. 2025 Apr 25;16:3873. doi: 10.1038/s41467-025-58907-y (PMC12032359; doi:10.1038/s41467-025-58907-y)
Supplement: Supplementary file 1 — Supplementary Information [file 41467_2025_58907_MOESM1_ESM.pdf]

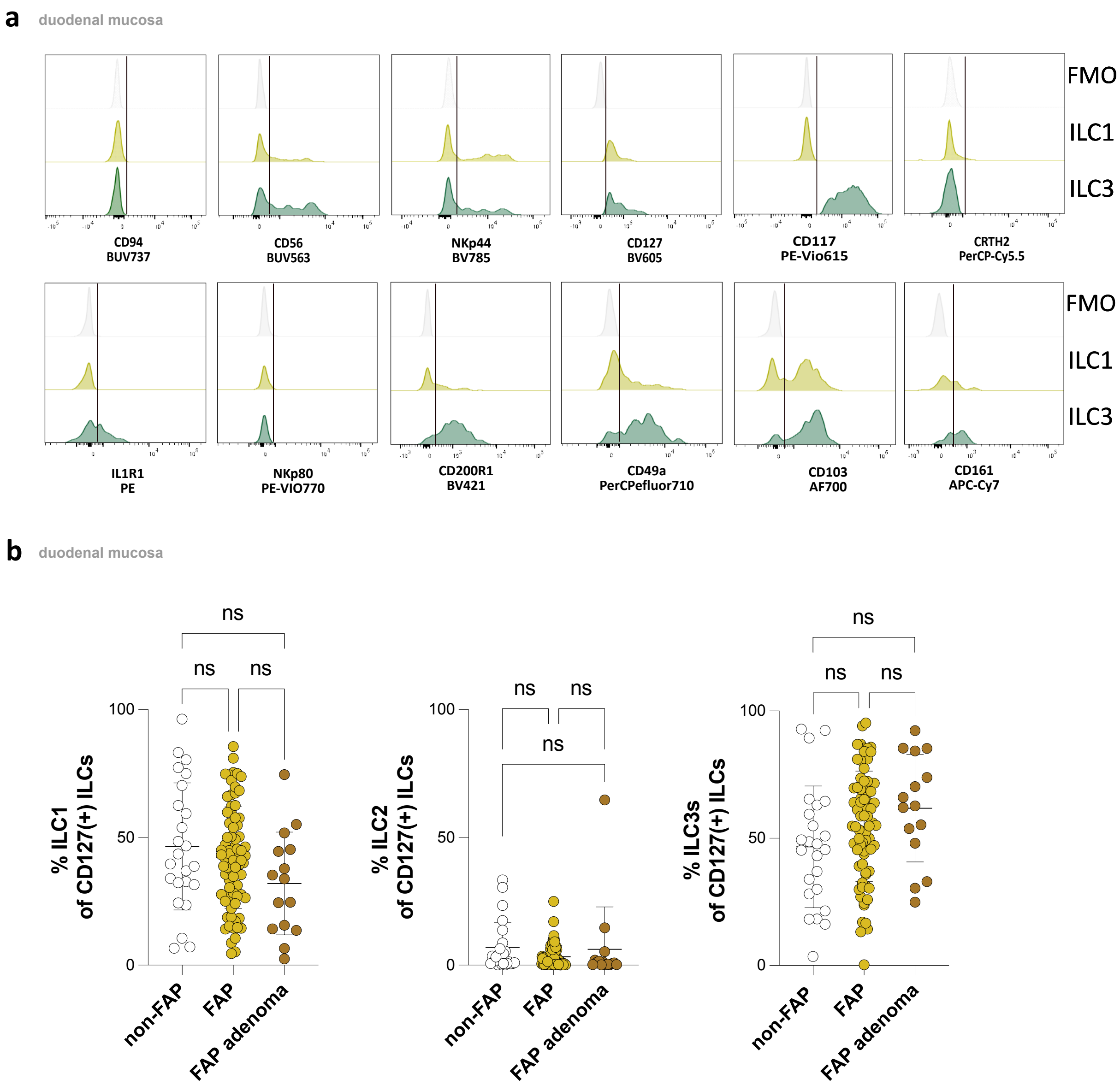

Supplementary Fig.1(related to Fig.1)

a Representative histograms showing ILC-specific markers for ILC1(yellow), ILC3(green) in duodenal tissue samples. Gating strategy of respective subsets in Fig. 1 and Supplementary Fig. 9b.

b Percentage of ILC1, ILC2, and ILC3 within total CD127(+) ILCs in duodenal adenomatous (n=15)(brown) and normal (n=76)(ochre) duodenal tissue of FAP patients, as well as normal duodenal mucosa (n=24) of non-FAP controls(white). Gating strategy of respective subsets in Fig. 1b. Mean ± SD.

Statistical significance analyzed by Kruskal-Wallis (KW) test (c) corrected for multiple comparisons using FDR (Benjamini, Krieger, Yekutieli).ns = not significant. All statistical tests are listed in the Source Data.

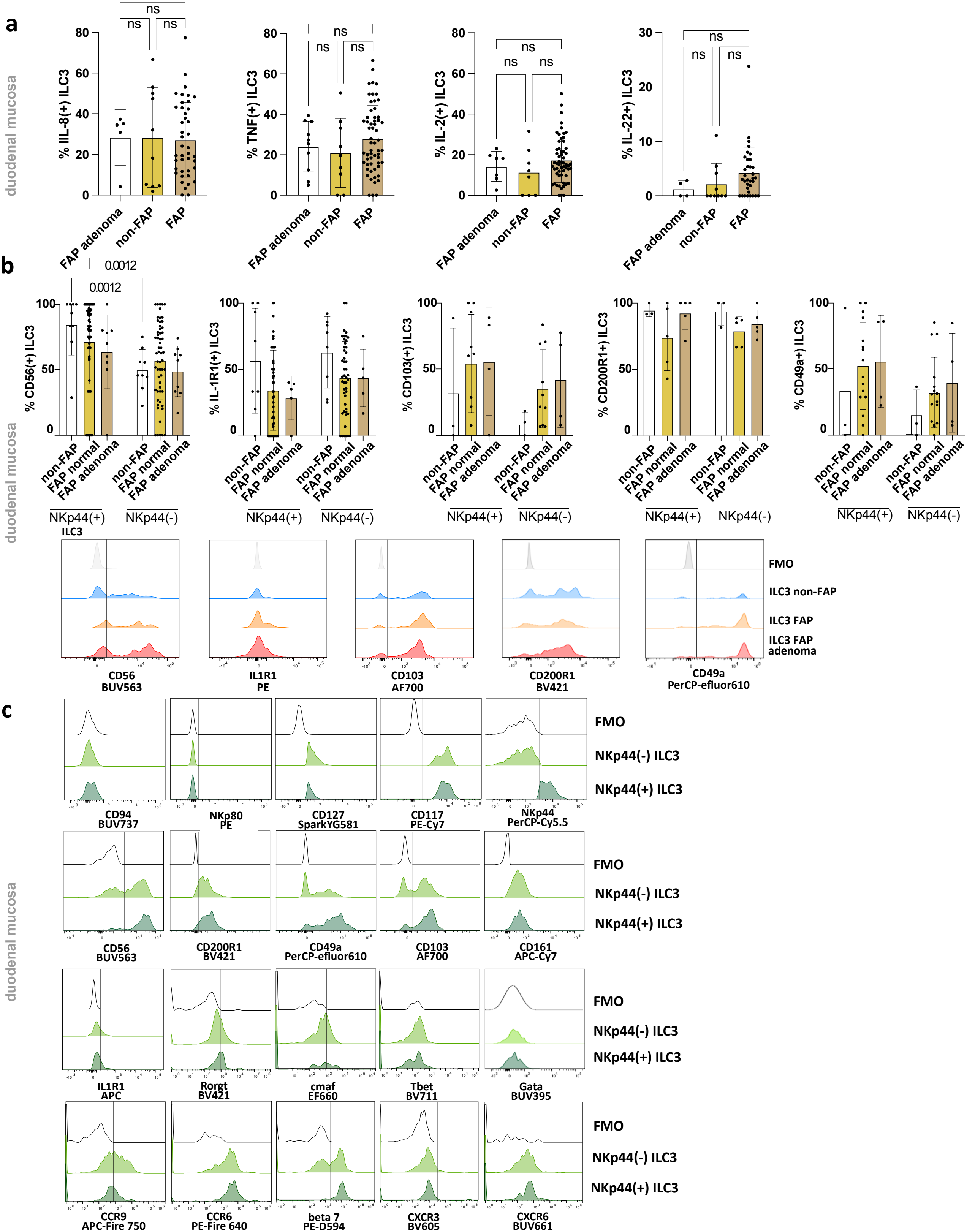
a
b
c
duodenal mucosa
% IIL-8(+) ILC3
% TNF(+) ILC3
% IL-2(+) ILC3
% IL-22+) ILC3
ns
FAP adenoma
non-FAP
FAP
0.0012
% CD56(+) ILC3
% IL-1R1(+) ILC3
% CD103(+) ILC3
% CD200R1+) ILC3
% CD49a+) ILC3
non-FAP
FAP normal
FAP adenoma
NKp44(+)
NKp44(-)
ILC3
FMO
ILC3 non-FAP
ILC3 FAP
ILC3 FAP adenoma
CD56 BUV563
IL1R1 PE
CD103 AF700
CD200R1 BV421
CD49a PerCP-efluor610
NKp44(-) ILC3
NKp44(+) ILC3
CD94 BUV737
NKp80 PE
CD127 SparkYG581
CD117 PE-Cy7
NKp44 PerCP-Cy5.5
CD56 BUV563
CD200R1 BV421
CD49a PerCP-efluor610
CD103 AF700
CD161 APC-Cy7
IL1R1 APC
Rorgt BV421
cmaf EF660
Tbet BV711
Gata BUV395
CCR9 APC-Fire 750
CCR6 PE-Fire 640
beta 7 PE-D594
CXCR3 BV605
CXCR6 BUV661

Supplementary Fig.2(related to Fig.2)

a Intracellular cytokine production of ILC-specific cytokines (IL-8, IL-2, TNF-α, IL-22) in CD127(+)ILC3 in duodenal adenomatous and normal mucosa of FAP patients and normal mucosa of non-FAP controls following PMA/Ionomycin stimulation. Mean ± SD. All case numbers are precisely listed in the Source Data for the respective comparisons. Gating strategy of respective subsets in Supplementary Fig. 9a.

b Percentages (upper panel) and representative stainings with FMO controls of indicated markers in a histogram (lower panel) of NKp44(+) and NKp44(-) cells among ILC3 in duodenal adenomatous (red) and normal mucosa of FAP patients(yellow), and normal mucosa of non-FAP patients(blue). Gating strategy of respective subsets in Supplementary Fig. 9b.

Mean ± SD. All case numbers are precisely listed in the Source Data for the respective comparisons. **c** Representative stainings of NKp44(+) and NKp44(-)ILC3 with indicated markers and FMO control. Gating strategy of respective subsets in Supplementary Fig. 9b/d. Statistical significance analyzed by Kruskal-Wallis (KW) test corrected for multiple comparisons using FDR (Benjamini, Krieger, Yekutieli). All statistical tests and the corresponding q-values are listed in the Source Data. Non-FAP are white, normal FAP are ochre and FAP adenomas are brown in the a & b(only upper panel) subdivisions diagrams.

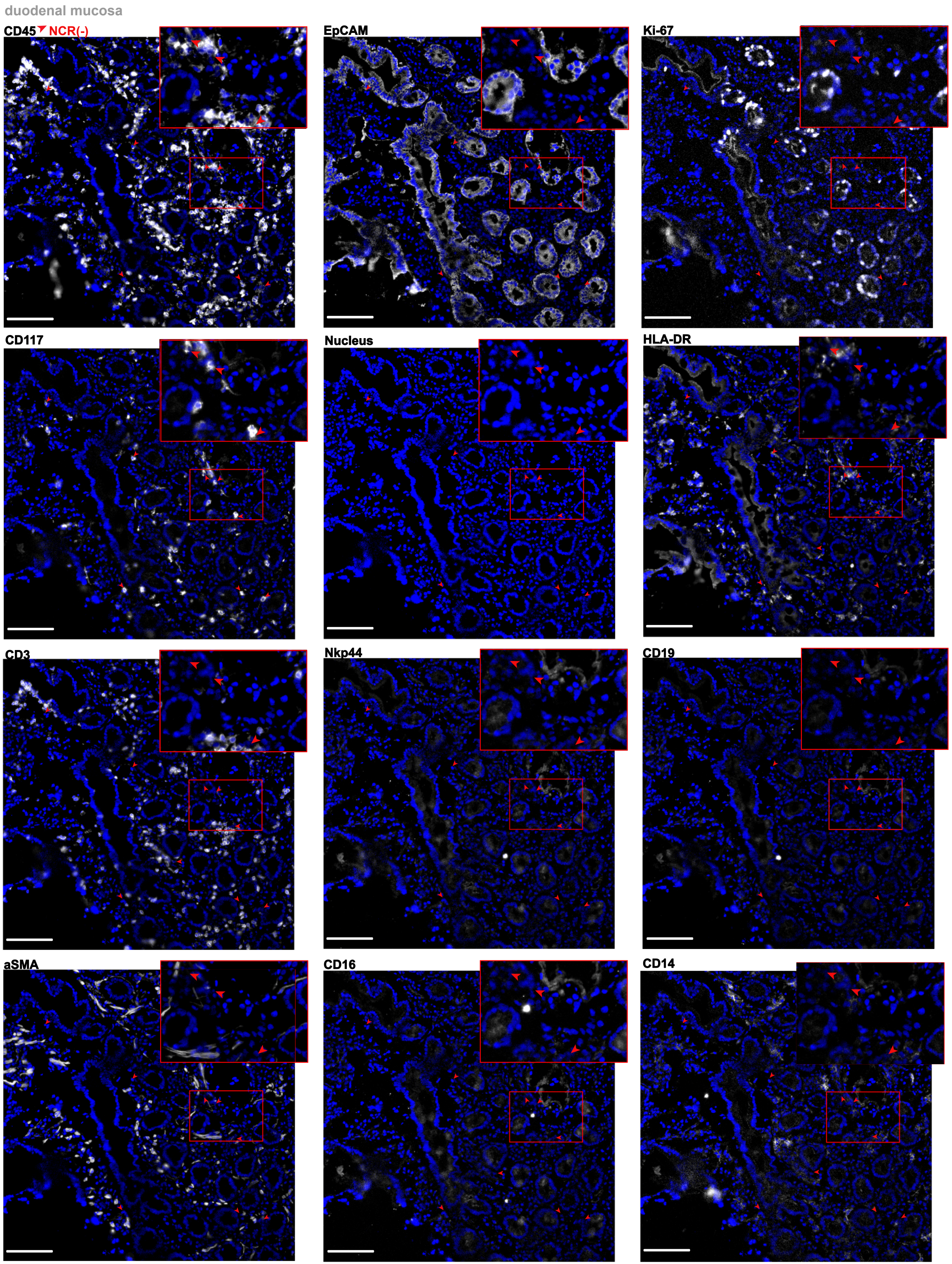

duodenal mucosa
CD45
NCR(-)
EpCAM
Ki-67
CD117
Nucleus
HLA-DR
CD3
Nkp44
CD19
aSMA
CD16
CD14

Supplementary Fig.3(related to Fig.3)

Representative MELC images showing individual stainings combined with nucleus staining. NKp44(-)ILC3 are defined as CD45(+)CD117(+)NKp44(-)CD3(-)CD14(-)CD16(-)CD19(-)EpCAM(-) lymphoid cells (red arrow). Additional markers include Ki-67, HLA-DR, and alpha-SMA. White scale bar represents 100 μm.

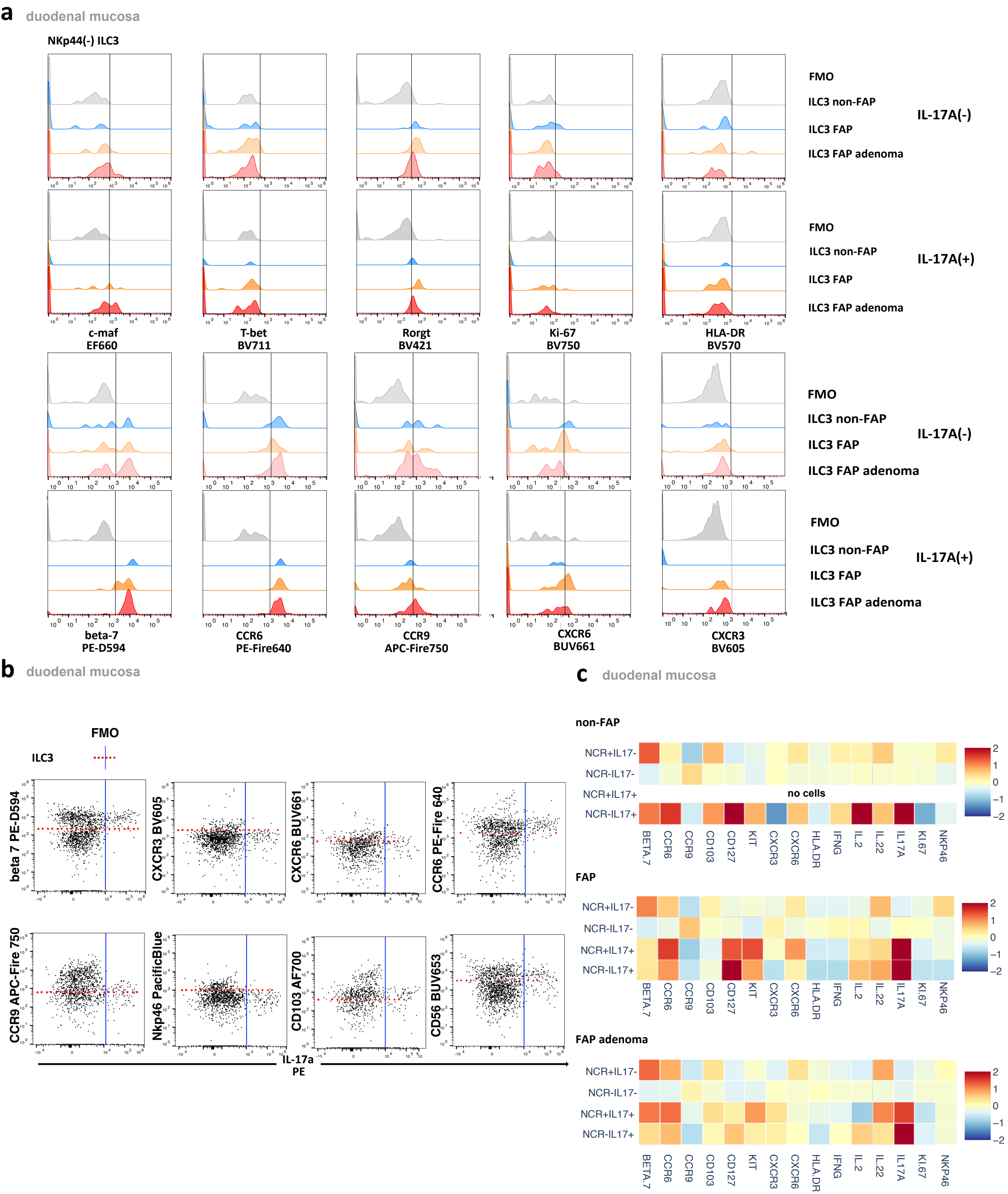
a
duodenal mucosa
NKp44(-) ILC3
FMO
ILC3 non-FAP
ILC3 FAP
ILC3 FAP adenoma
IL-17A(-)
IL-17A(+)
c-maf
EF660
T-bet
BV711
Rorgt
BV421
Ki-67
BV750
HLA-DR
BV570
beta-7
PE-D594
CCR6
PE-Fire640
CCR9
APC-Fire750
CXCR6
BUV661
CXCR3
BV605
b
duodenal mucosa
FMO
ILC3
beta 7 PE-D594
CXCR3 BV605
CXCR6 BUV661
CCR6 PE-Fire 640
CCR9 APC-Fire 750
Nkp46 PacificBlue
CD103 AF700
CD56 BUV653
IL-17a
PE
c
duodenal mucosa
non-FAP
FAP
FAP adenoma
NCR+IL17-
NCR-IL17-
NCR+IL17+
NCR-IL17+
no cells
BETA.7
CCR6
CCR9
CD103
CD127
KIT
CXCR3
CXCR6
HLA.DR
IFNG
IL.2
IL.22
IL17A
KI.67
NKP46

Supplementary Fig.4(related to Fig.3)

a Representative stainings of NKp44(-)ILC3, split into IL-17A(-) and IL-17A(+) subsets, with indicated markers and FMO controls, following PMA/Ionomycin stimulation. Gating strategy of respective subsets in Supplementary Fig. 9d.

b Dot plot showing co-expression of IL-17A of ILC3 cells with indicated markers, including FMO cut-off, following PMA/Ionomycin stimulation. Gating strategy of respective subsets in Supplementary Fig. 9d.

c Heatmaps showing z-score of flow cytometric evaluated expressions of indicated markers, gated on NKp44(+) and NKp44(-)ILC3s following PMA/Ionomycin stimulation, split into IL-17(-) and IL-17(+) subsets, and further divided into non-FAP, normal FAP, and FAP adenoma groups, from three subjects each(blue-yellow-red scaling).

FMO (Fluorescence minus One) control (grey), Non-FAP are blue, normal FAP are yellow and FAP adenomas are red in the a subdivisions histograms. Gating strategy of respective subsets in Supplementary Fig. 9d.

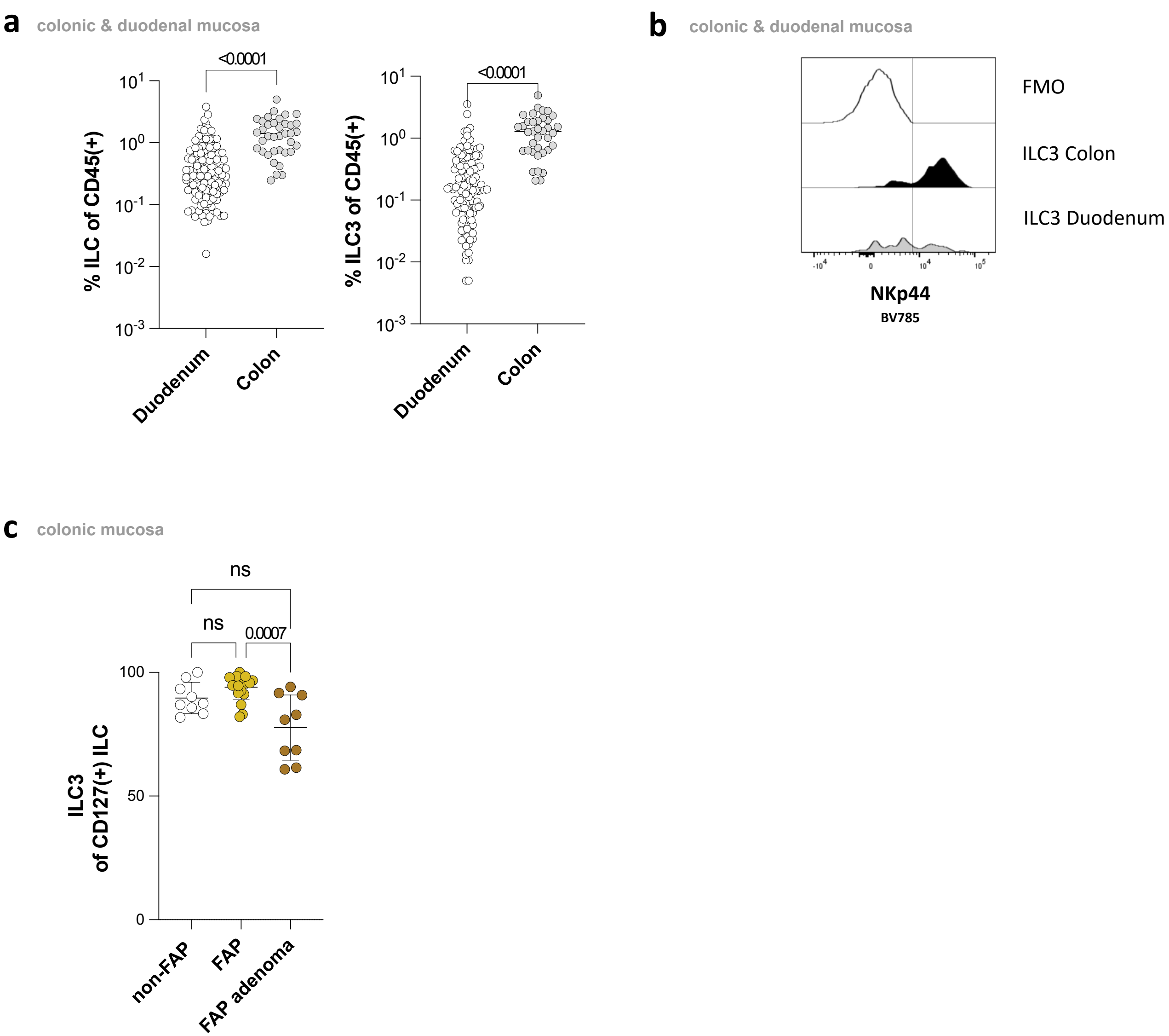

Supplementary Fig.5(related to Fig.4)

a Frequency of total ILCs and ILC3s of CD45(+) cells in the duodenum (n=115) compared to the colon (n=37). Mean is displayed in the center. Gating strategy of respective subsets in Fig. 1b.

b Representative histogram displaying NKp44 expression of colon and duodenum ILC3 with FMO(Fluorescence minus One) control. Gating strategy of respective subsets in Fig. 1b.

c Percentages of colonic ILC3s in colonic adenomatous (n=9)(brown) and normal mucosa (n=19)(ochre) of FAP patients and normal mucosa (n=9) of non-FAP controls(white). Gating strategy of respective subsets in Fig. 1b. Mean ± SD. Statistical significance analyzed by Two-tailed Mann-Whitney test (a) and Kruskal-Wallis (KW) test (c) corrected for multiple comparisons using FDR (Benjamini, Krieger, Yekutieli). *q≤0.05; ****p≤0.0001; ns = not significant. All statistical tests are listed in the Source Data.

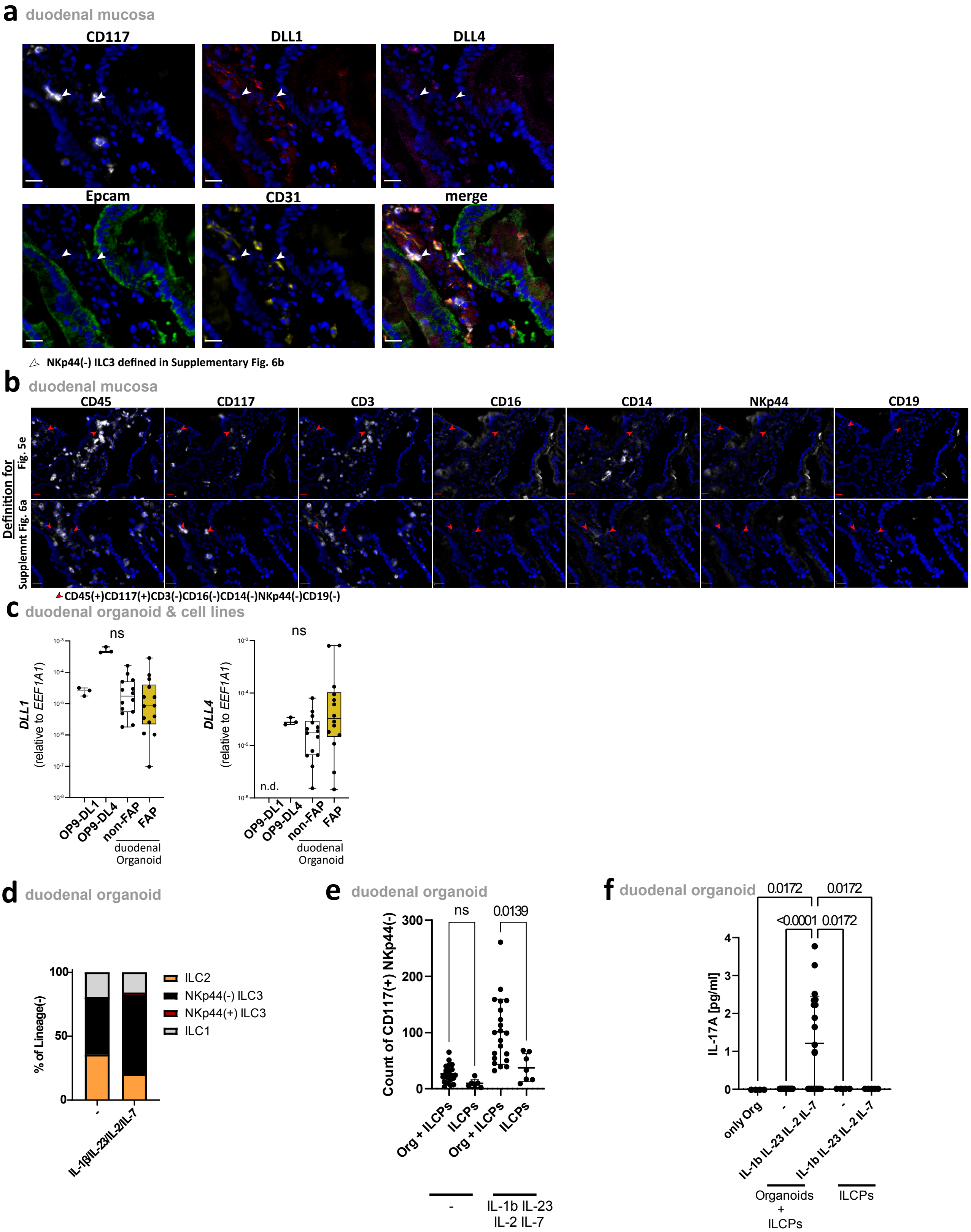

a duodenal mucosa
CD117
DLL1
DLL4
Epcam
CD31
merge
NKp44(-) ILC3 defined in Supplementary Fig. 6b
b duodenal mucosa
CD45
CD117
CD3
CD16
CD14
NKp44
CD19
Definition for Fig. 5e
Supplemnt Fig. 6a
CD45(+)CD117(+)CD3(-)CD16(-)CD14(-)NKp44(-)CD19(-)
c duodenal organoid & cell lines
ns
DLL1 (relative to EEF1A1)
OP9-DL1
OP9-DL4
non-FAP
FAP
duodenal Organoid
ns
DLL4 (relative to EEF1A1)
n.d.
OP9-DL1
OP9-DL4
non-FAP
FAP
duodenal Organoid
d duodenal organoid
% of Lineage(-)
100
50
0
ILC2
NKp44(-) ILC3
NKp44(+) ILC3
ILC1
-
IL-1β/IL-23/IL-2/IL-7
e duodenal organoid
Count of CD117(+) NKp44(-)
300
200
100
0
ns
0.0139
Org + ILCPs
ILCPs
Org + ILCPs
ILCPs
-
IL-1b IL-23
IL-2 IL-7
f duodenal organoid
0.0172
0.0172
<0.0001
0.0172
IL-17A [pg/ml]
4
3
2
1
0
only Org
-
IL-1b IL-23 IL-2 IL-7
-
IL-1b IL-23 IL-2 IL-7
Organoids + ILCPs
ILCPs

Supplementary Fig.6(related to Fig.5)

a Representative MELC images of FAP normal tissue showing NKp44(-)ILC3 cells, indicated by white arrows, with CD117(white), DLL1(red), DLL4(violet), EpCAM(green), CD31(yellow) and nucleus stainings(blue). NKp44(-)ILC3 cells are defined in Supplementary Fig.7F. White scale bar represents 20 μm. **b** Representative MELC images showing individual stainings combined with nucleus staining. NKp44(-)ILC3 are defined as CD45(+)CD117(+)NKp44(-)CD3(-)CD14(-)CD16(-)CD19(-)EpCAM(-) lymphoid cells (red arrow). EpCAM is visibly defined as negative in Fig.5e and Supplementary Fig.6b, respectively. The upper row pertains to Fig.4J, and the lower row pertains to Supplementary Fig.7E. Red scale bar represents 20 μm. **c** *DLL1* and DLL4 mRNA expression in OP9-DL1(n=3), OP9-DL4(n=3), non-FAP(n=14)(white) and FAP(n=14)(ochre) derived duodenal organoids. mRNA expression levels are relative to *EEF1A1* expression. Box plot showing the median (center line), interquartile range (box bounds: 25th–75th percentile), and range (whiskers: min–max). **d** Percentages of ILC1(grey), ILC2(orange), NKp44(+)ILC3(red), and NKp44(-)ILC3(black) within matrigel dome containing duodenal organoids from FAP patients after 7 days of culturing ILCPs under different conditions. **e** Count of CD117(+) NKp44(-) ILCs within matrigel dome containing duodenal organoids from FAP patients or only cells after 7 days of culturing ILCPs under different conditions. Precise case numbers are listed in the Source Data. Mean ± SD. **f** IL-17A concentration in the supernatant of ILCP/organoid after 7 days of co-culture and controls. Precise case numbers are listed in the Source Data. Mean ± SD. Statistical significance analyzed by Kruskal-Wallis (KW) test corrected for multiple comparisons using FDR (Benjamini, Krieger, Yekutieli). ns = not significant. All statistical tests are listed in the Source Data.

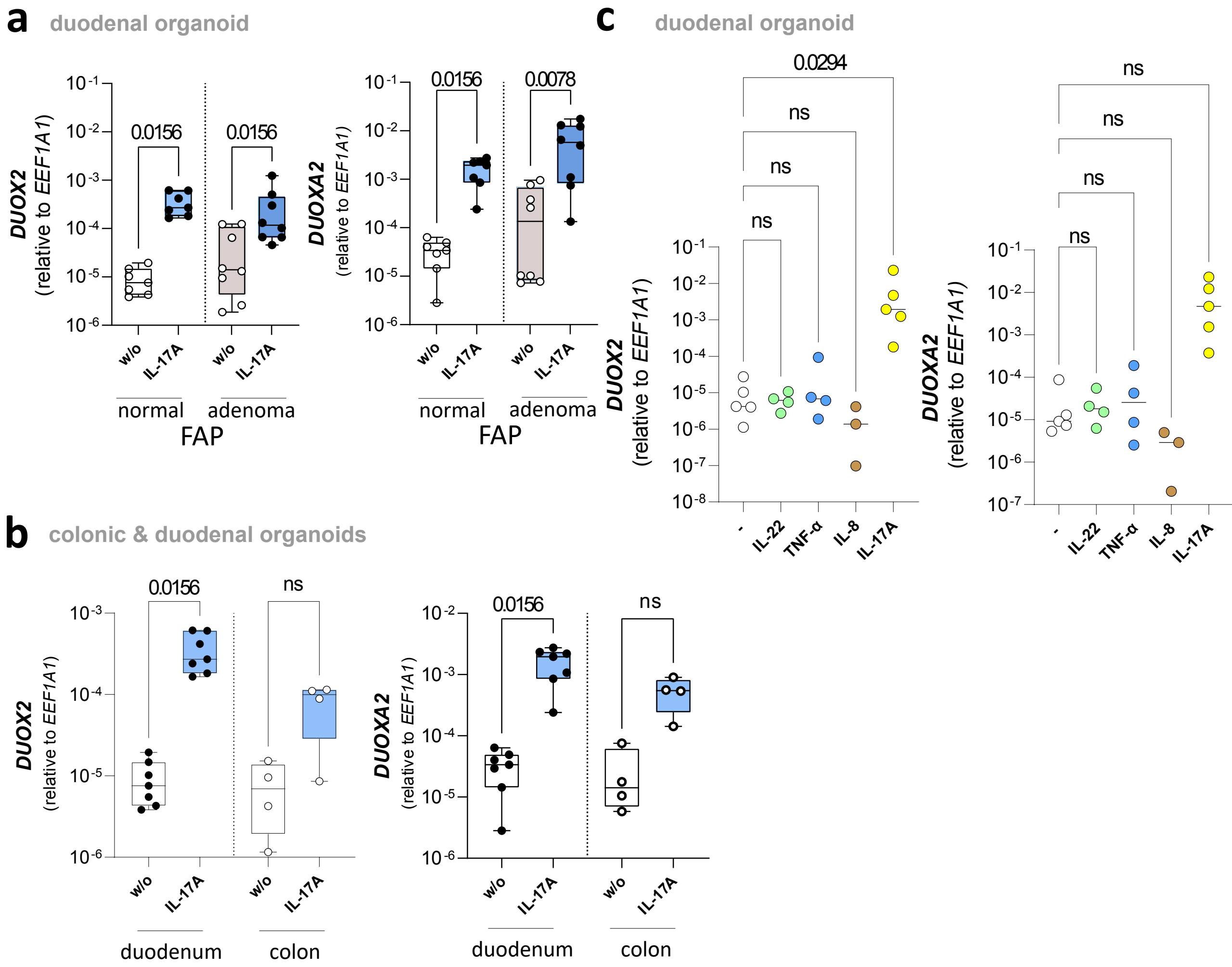

Supplementary Fig.7(related to Fig.7)

a *DUOX2* and *DUOXA2* mRNA expression in duodenal organoids established from FAP normal (n=5) and FAP adenomatous (n=3) tissue cultured in the presence (blue) or absence (white for normal FAP or grey for FAP adenoma) of IL-17A. Box plot showing the median (center line), interquartile range (box bounds: 25th–75th percentile), and range (whiskers: min–max).

b *DUOX2* and *DUOXA2* mRNA expression in duodenal (n=5) and colonic (n=3) organoids in the presence(blue) or absence(white) of IL-17A. Box plot showing the median (center line), interquartile range (box bounds: 25th–75th percentile), and range (whiskers: min–max).

c *DUOX2* and *DUOXA2* mRNA expression in duodenal organoids cultured in the absence (n=5) or presence of IL-22 (n=4)(green), TNF-α (n=4)(blue), IL-8 (n=3)(brown), or IL-17A (n=5)(yellow). mRNA expression levels are relative to *EEF1A1*. Mean is displayed in the center.

Statistical significance analyzed by Wilcoxon matched-pairs signed rank test (a,b) and by Kruskal-Wallis (KW) test (c) corrected for multiple comparisons using FDR (Benjamini, Krieger, Yekutieli). ns = not significant. All statistical tests are listed in the Source Data.

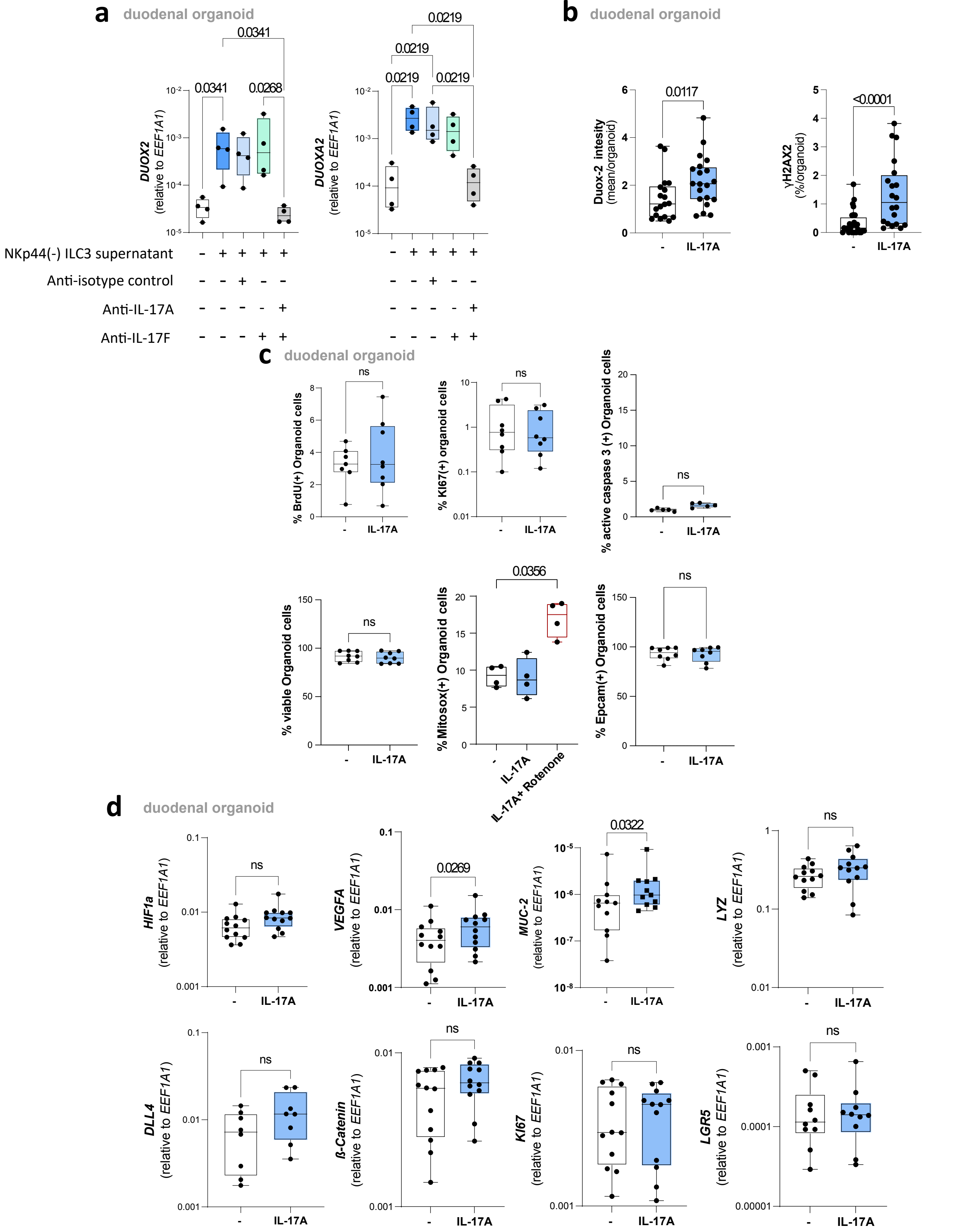

a
duodenal organoid
DUOX2 (relative to EEF1A1)
0.0341
0.0341
0.0268
DUOXA2 (relative to EEF1A1)
0.0219
0.0219
0.0219
0.0219
NKp44(-) ILC3 supernatant
Anti-isotype control
Anti-IL-17A
Anti-IL-17F
b
duodenal organoid
Duox-2 intesity (mean/organoid)
0.0117
γH2AX2 (%/organoid)
<0.0001
IL-17A
c
duodenal organoid
% BrdU(+) Organoid cells
% KI67(+) organoid cells
% active caspase 3 (+) Organoid cells
% viable Organoid cells
% Mitosox(+) Organoid cells
0.0356
% Epcam(+) Organoid cells
ns
IL-17A
IL-17A+ Rotenone
d
duodenal organoid
HIF1a (relative to EEF1A1)
VEGFA (relative to EEF1A1)
0.0269
MUC-2 (relative to EEF1A1)
0.0322
LYZ (relative to EEF1A1)
DLL4 (relative to EEF1A1)
ß-Catenin (relative to EEF1A1)
KI67 (relative to EEF1A1)
LGR5 (relative to EEF1A1)
ns
IL-17A

Supplementary Fig.8(related to Fig.8)

a *DUOX2* and *DUOXA2* mRNA levels were measured in duodenal organoid cultures (n=4) incubated with or without NKp44(-)ILC3 supernatants in the presence or absence of an IL-17A or IL-17F-specific blocking antibody as indicated (control: white; NKp44(-)ILC supernatants only: blue; supernatant with Isotype control: light blue; supernatant with anti-IL-17F: green; supernatant with anti-IL-17A: grey). **b** Left panel: Duox2 mean intensity relative to Hoechst staining in unstimulated (n=18) and IL-17A-stimulated duodenal organoid cultures (n=18). Right panel: frequency of γH2AX(+) cells in unstimulated duodenal organoids (n=28) and IL-17A-stimulated organoid cultures (n=20), with a stimulation time of 1 day for both detections. **c** Flow cytometric detection of duodenal organoid cultures after 5-day with IL-17A or no stimulation, analyzing % positive expression for BrdU (n=7), Ki-67 (n=8), active caspase 3 (n=5), viability (n=8), Mitosox (n=4; with Rotenone as Mitosox positive control), and Epcam (n=8). **d** mRNA levels were measured in duodenal organoids cultures (n≥10) after 5-day with IL-17A or no stimulation. mRNA expression levels are relative to *EEF1A1* expression. Box plots in all subdivisions showing the median (center line), interquartile range (box bounds: 25th–75th percentile), and range (whiskers: min–max).

Statistical significance analyzed by Wilcoxon matched-pairs signed rank test (c,d), Mann-Whitney-Test (b), and Friedman test (a) corrected for multiple comparisons using FDR (Benjamini, Krieger and Yekutieli). ns = not significant. All statistical tests are listed in the Source Data. In subdivision b,c & d, non-stimulated bars are white, IL-17A stimulated bars are blue, and rotenone-stimulated IL-17A is white bar combined with a red line

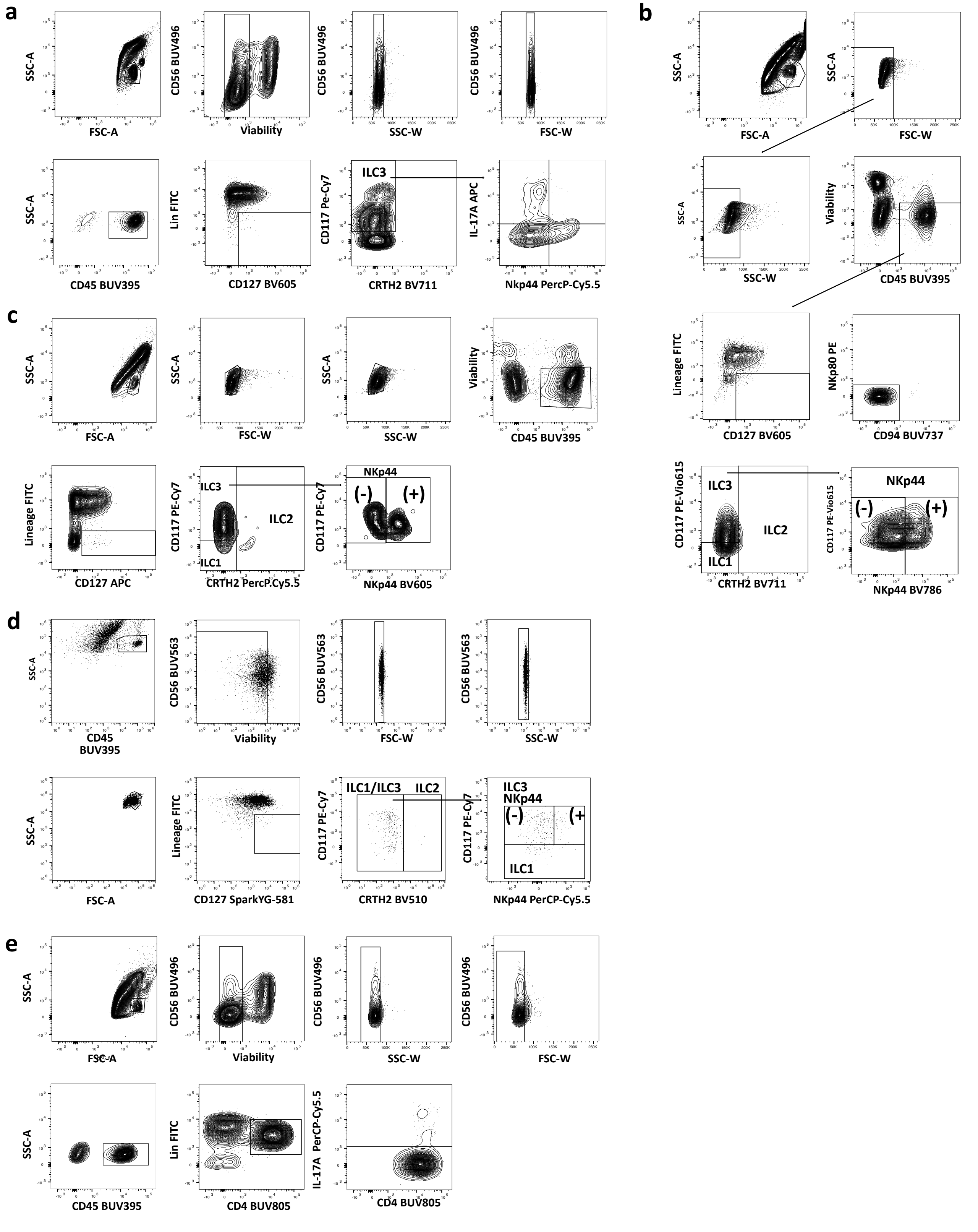
a
SSC-A
FSC-A
CD56 BUV496
Viability
SSC-W
FSC-W
CD45 BUV395
Lin FITC
CD127 BV605
ILC3
CD117 Pe-Cy7
CRTH2 BV711
IL-17A APC
Nkp44 PercP-Cy5.5
b
Viability
Lineage FITC
NKp80 PE
CD94 BUV737
ILC3
ILC2
ILC1
CD117 PE-Vio615
CRTH2 BV711
NKp44
(-)
(+)
NKp44 BV786
c
CD127 APC
CRTH2 PercP.Cy5.5
NKp44 BV605
d
CD56 BUV563
CD45
BUV395
CD127 SparkYG-581
ILC1/ILC3
CRTH2 BV510
NKp44 PerCP-Cy5.5
e
CD4 BUV805
IL-17A PerCP-Cy5.5

Supplementary Fig.9(related to Fig.2/3/4/5 and Supplementary Fig.4/6)

a Representative FACS plots showing gating strategy for intestinal lymphocytes to NKp44(-) and NKp44(+) ILC3 cells (related to Fig. 2). Lineage for this panel is defined as CD3, CD4, CD5, CD14, CD19, CD20, TCRγδ, TCRαβ, BDCA-2, CD1a, CD34, NKp80, CD94, FcεR1a and CD123. **b** Representative FACS plots showing gating strategy for intestinal lymphocytes to NKp44(-) and NKp44(+) ILC3 cells (related to Supplementary Fig. 2). CD3, CD4, CD5, CD14, CD19, CD20, TCRγδ, TCRαβ, BDCA-2, CD1a, CD34, FcεR1a and CD123. NKp80& CD94 were excluded. **c** Representative FACS plots showing sorting strategy for intestinal or tonsillar lymphocytes to ILC1, NKp44(-) and NKp44(+) ILC3 cells (related to Fig. 2/5 and Supplementary Fig.6). Lineage for this panel is defined as CD3, CD4, CD5, CD14, CD19, CD20, TCRγδ, TCRαβ, BDCA-2, CD1a, CD34, NKp80, CD94, FcεR1a and CD123. **d** Representative FACS plots showing gating strategy for intestinal lymphocytes to NKp44(-) and NKp44(+) ILC3 cells (related to Fig. 3 and Supplementary Fig. 2/4).Lineage for this panel is defined as CD3, CD4, CD5, CD94, NKp80, CD14, CD19, CD20, TCRγδ, TCRαβ, BDCA-2, CD1a, CD34, FcεR1a and CD123. **e** Representative FACS plots showing gating strategy for intestinal lymphocytes to CD4(+)Lin(+) cells (related to Fig. 3). Lineage for this panel is defined as CD3, CD14, CD19, CD20, TCRγδ, TCRαβ, BDCA-2, CD1a, CD34, NKp80, CD94, FcεR1a and CD123.

Supplementary Table 1: Consumables

Component	Manufacturer	Order No.
2-Mercaptoethanol	Sigma-Aldrich®	M6250-100ML
Antibiotic-Antimycotic	Gibco™	15240062
Ascorbic acid	European Pharmacopoeia	A1300000
Brefeldin A (BFA)	Enzo	BML-G405
BSA	Sigma Aldrich®	A9418
Collagenase Type IV	Worthington®	LS004189
Corning® Cell Recovery Solution	Corning	354253
DTT	Sigma-Aldrich®	10708984001
EDTA	AppliChem	A3145
Fetal Bovine Serum (heat-inactivated)	Sigma-Aldrich®	Cat#F7524-
Gentle Dissociation Reagent	StemCell™ Technologies	100-0485
goat serum	Sigma-Aldrich®	S26
Ham's F-12 Nutrient Mix	Gibco™	21765029
Hanks' Balanced Salt Solution (HBSS)	Gibco™	14180046
Hoechst 33342 Fluorescent Stain	Thermo Fisher Scientific	62249
HS-Nuclease	MoBiTec®	GE-NUC10700
Human AB serum	Sigma-Aldrich®	H3667-100ML
IntestiCult™ Organoid Growth Medium (Human)	StemCell™ Technologies	# 06010
Ionomycin	Cell Signaling Technology Europe	9995
LS MACS colums	Miltenyi Biotec	130-042-401
Matrigel	Corning™	356237
NAC	Sigma-Aldrich®	A7250
Pancoll	PanBiotec™	P04-601000
PBS	Gibco™	18912014
Penicillin-Streptomycin	PanBiotec™	P06-07050
PFA	Merck	158127
Phorbol-12-myristat-13-acetat (PMA)	Cell Signaling Technology Europe	9905
Prolong™ Gold antifade reagent	Invitrogen™	P36930
RPMI-1640	Gibco™	21875034
sodium acid	Merck KGaA	26628-22-8
Sodium selenite	Sigma-Aldrich®	S5261-10G
Triton-X100	Merck KGaA	9036-19-5
TrypLE	Thermo Fisher Scientific	12604021
MACS BSA Stock Solution	Miltenyi Biotec	130-091-376
**Hardware**	**Manufacturer**	**Order No.**
MACS® SmartStrainers (70 µm)	Miltenyi Biotec	130-110-916
24-Well culture plate	Greiner Bio-One	662102
24-Well suspensions culture plate	Greiner Bio-One	662160
96-Well F-bottom culture plate	Greiner Bio-One	655180
Microplate 96-Well V-plate	Greiner Bio-One	651201
96-Well U-bottom culture plate	Greiner Bio-One	650180
**Kit**	**Manufacturer**	**Order No.**
active caspase 3	BD Pharmingen	**550914**
Blue S' Green qPCR Kit	Biozym®	331416
BrdU	BD Pharmingen	556405
Cytofix/Cytoperm™ Fixation/Permeablization Kit	BD Bioscience	554714
DCFDA / H2DCFDA - Cellular ROS Assay Kit	Abcam	ab113851
eBioscience™ Foxp3 / Transcription Factor Staining	Invitrogen™	00-5523-00
GeneJet RNA purification kit	Thermo Scientific	K0732
LEGENDplex™ Human Th Cytokine Panel kit	Biolegend®	740001
LIVE/DEAD™ Kit	Invitrogen™	L34975
MitoSOX™	Thermo Scientific	#M36008
QuantiTect® Reverse Transcription Kit	Qiagen	205313
Zombie Aqua™ Fixable Viability Kit	Biolegend®	423102
Zombie NIR	Biolegend®	423106
UltraView / OptiView DAB detection kit	Roche	760-700
UltraView Universal Alkaline Phosphatase Red	Roche	760-501
**Cytokines**	**Manufacturer**	**Order No.**
IL-17A	Biolegend	570504
IL-1β	ImmunoTools	11340015
IL-2	Miltenyi Biotec	130-097-743
IL-2	Miltenyi Biotec	130-097-743
IL-22	Immunotools	11340223
IL-23	ImmunoTools	11340233
IL-7	Miltenyi Biotec	130-095-367
IL-8	Immunotools	11349084
TNF-α	Immunotools	11343015
sDLL1	PeproTech	140-08
SDLL4	PeproTech	140-07

Supplementary Table 2: Media

**Pre-Digestion Medium**	
HBSS	
DTT	154 µg/ml
EDTA	5 mM
NAC	0.25 %
P/S	1 %

**Digestion Medium**	
RPMI-1640	
FBS	10 %
P/S	1 %
Collagenase Type IV	125 U/ml
HS-Nucelase	25 U/ml

**Freezing Medium**	
RPMI-1640	
FBS	10 %
DMSO	10 %
P/S	1 %

**Thawing Medium**	
RPMI-1640	
FBS	10 %
P/S	1 %
HS-Nucelase	25 U/ml

**complete RPMI**	
RPMI-1640	
FBS	10 %
P/S	1 %

**Differentiation medium (OP9 Bulk Culture)**	
Ham's F12 Nutrient Mix	
Human AB Serum	10 %
Antibiotic-Antimycotic	1 %
Ascorbic acid	20 mg/ml
Sodium selenite	0.05 mg/ml
2-Mercaptoethanol	24 mM

**Blocking Buffer**	
DPBS	
goat serum	5 %
BSA	1 %
Triton-X	0.1 %
sodium acid	0.04 %

**Antibody buffer**	
DPBS	
BSA	0,01
Triton-X	0.1 %
sodium acid	0.04 %

MELC washing solution	
PBS	
MACS BSA	5 %
Triton X-100	0.02 %

Supplementary Table 3: Primer

Gene Name	Fwd Primer 5'-3' Seq	Rev Primer 5'-3' Seq	Amplicon Size [bp]
CAPN8	GACTTCCAGGAGAACTATGCGG	TCCGAGTGTAGGAAGAGCAGCT	125
CDH3	CAGGTGCTGAACATCACGGACA	CTTCAGGGACAAGACCACTGTG	135
CEMIP	ACCGAGCACATTCCAACTACCG	GGCAGAGATGATTGAGAGGAACG	107
DLL1	GATTCTCCTGATGACCTCGCA	TCCGTAGTAGTGTTCGTCACA	168
DLL4	GTCTCCACGCCGGTATTGG	CAGGTGAAATTGAAGGGCAGT	98
DUOX2	CTGGGTCCATCGGGCAATC	GTCGGCGTAATTGGCTGGTA	144
DUOXA2	AACGGCGTACTGCCTTTTTAC	GAGAAGAACTCTCACCAACCAAA	165
EEF1A1	CCG TTC TTC CAC CAC TGA TT	CTT TGG GTC GCT TTG CTG TT	183
IL1B	GAA GCT GAT GGC CCT AAA CA	AAG CCC TTG CTG TAG TGG TG	110
IL23A	CTC AGG GAC AAC AGT CAG TTC	ACA GGG CTA TCA GGG AGC A	119
KI67	TCCTTTGGTGGGCACCTAAGACCTG	TGATGGTTGAGGTCGTTCCTTGATG	156
LGR5	CCTGCTTGACTTTGAGGAAGACC	CCAGCCATCAAGCAGGTGTTCA	100
LYZ	TCAATAGCCGCTACTGGTGTA	ATCACGGACAACCCTCTTTGC	131
MUCIN-2	GGAGATCACCAATGACTGCGA	GAATCGTTGTGGTCACCCTTG	183
S100P	CTCAAGGTGCTGATGGAGAAGG	GAACTCACTGAAGTCCACCTGG	126
TSPAN1	TGCTGTGGTCGCCTTGGTGTAC	TGGTGAAGCCACAGCACTTGAG	152
HIF1a	TATGAGCCAGAAGAACTTTTAGGC	CACCTCTTTTGGCAAGCATCCTG	145
VEGFA	TTGCCTTGCTGCTCTACCTCCA	GATGGCAGTAGCTGCGCTGATA	126
ß-Catenin	CACAAGCAGAGTGCTGAAGGTG	GATTCCTGAGAGTCCAAAGACAG	146

Supplementary Table 4: tools & software

Tool/Software	Version	Usage	Source
Microsoft Office	2016 & 395	all data	https://www.microsoft.com/de-de/microsoft-365/
GraphPad Prism	9.4.0 & 10.4.1	all data	https://www.graphpad.com/features
R	3.6.2 or 4.0.3 or 4.2.0	bulk RNASeq	https://www.r-project.org/about.html
ggplot	3.3.2	bulk RNASeq	https://github.com/tidyverse/ggplot2
complexheatmap	2.2.0	bulk RNASeq	https://github.com/jokergoo/ComplexHeatmap
DESeq2	v1.38.1	bulk RNASeq	https://bioconductor.org/packages/release/bioc/html/DESeq2.html
MSigDBdatabases	n.a.	bulk RNASeq	https://www.gsea-msigdb.org/gsea/msigdb
bcl2fastq2	2.20	bulk RNASeq	https://support.illumina.com/downloads/bcl2fastq-conversion-software-v2-20.html
nf-core rnaseqpipeline	3.11.2	bulk RNASeq	https://nf-co.re/rnaseq/3.14.0/
FASTQ	n.a.	bulk RNASeq	https://www.bioinformatics.babraham.ac.uk/projects/fastqc
MultiQC	n.a.	bulk RNASeq	https://github.com/MultiQC/MultiQC
kallisto	0.5	bulk RNASeq	https://github.com/pachterlab/kallisto
FlowJo	n.a.	Flowcytometry	https://www.flowjo.com
cytolytics	n.a.	Flowcytometry	https://cytolytics.de
ImageJ	1.2	Microscopy	https://imagej.net/ij/
Qupath 0.5.	0.5	Microscopy	https://qupath.readthedocs.io/en/0.5/
Zeiss software ZEN	3.6	Microscopy	https://www.zeiss.com/microscopy/de/produkte/software/zeiss-zen.html
LightCycler 96 software	1.1.0.1320	PCR	https://www.roche.de/diagnostik/produkte-loesungen/systeme/lightcycler-systeme#d8225de1-a897-4dea-a860-0d7115b3a985
n.a. not available			
