# Supplementary material for: IL-17A-producing NKp44(−) group 3 innate lymphoid cells accumulate in Familial Adenomatous Polyposis duodenal tissue
Source: Nat Commun. 2025 Apr 25;16:3873. doi: 10.1038/s41467-025-58907-y (PMC12032359; doi:10.1038/s41467-025-58907-y)
Supplement: Supplementary file 2 — Description of Additional Supplementary Files [file 41467_2025_58907_MOESM2_ESM.pdf]

Description of Additional Supplementary Files

Supplementary Data 1. Subject characteristics from FAP and non-FAP subjects.

Supplementary Data 2. Transcriptomic data of bulk RNASeq analysis (counts, DEGs, pathways, methods)

Supplementary Data 3. List of all antibodies (Flow Cytometry, IHC/Blocking, MELC).
