# Supplementary material for: IL-17A-producing NKp44(−) group 3 innate lymphoid cells accumulate in Familial Adenomatous Polyposis duodenal tissue
Source: Nat Commun. 2025 Apr 25;16:3873. doi: 10.1038/s41467-025-58907-y (PMC12032359; doi:10.1038/s41467-025-58907-y)
Supplement: Supplementary file 6 — Reporting Summary [file 41467_2025_58907_MOESM6_ESM.pdf]

nature portfolio

Corresponding author(s): Dr. Jacob Nattermann, Dr. Benjamin Krämer

Last updated by author(s): Jan 26, 2025

Reporting Summary

Nature Portfolio wishes to improve the reproducibility of the work that we publish. This form provides structure for consistency and transparency in reporting. For further information on Nature Portfolio policies, see our Editorial Policies and the Editorial Policy Checklist.

Statistics

For all statistical analyses, confirm that the following items are present in the figure legend, table legend, main text, or Methods section.

n/a	Confirmed	
☐	☒	The exact sample size (*n*) for each experimental group/condition, given as a discrete number and unit of measurement
☐	☒	A statement on whether measurements were taken from distinct samples or whether the same sample was measured repeatedly
☐	☒	The statistical test(s) used AND whether they are one- or two-sided<br>*Only common tests should be described solely by name; describe more complex techniques in the Methods section.*
☐	☒	A description of all covariates tested
☐	☒	A description of any assumptions or corrections, such as tests of normality and adjustment for multiple comparisons
☐	☒	A full description of the statistical parameters including central tendency (e.g. means) or other basic estimates (e.g. regression coefficient) AND variation (e.g. standard deviation) or associated estimates of uncertainty (e.g. confidence intervals)
☐	☒	For null hypothesis testing, the test statistic (e.g. *F*, *t*, *r*) with confidence intervals, effect sizes, degrees of freedom and *P* value noted<br>*Give P values as exact values whenever suitable.*
☒	☐	For Bayesian analysis, information on the choice of priors and Markov chain Monte Carlo settings
☐	☒	For hierarchical and complex designs, identification of the appropriate level for tests and full reporting of outcomes
☐	☒	Estimates of effect sizes (e.g. Cohen's *d*, Pearson's *r*), indicating how they were calculated

Our web collection on statistics for biologists contains articles on many of the points above.

Software and code

Policy information about availability of computer code

Data collection	bcl2fastq2, FASTQ, Zeiss software ZEN, LightCycler 96 software
Data analysis	Microsoft Office, GraphPad Prism, R, ggplot, complexheatmap, DESeq2, MSigDB databases, nf-core rnaseq pipeline, MultiQC, kallisto, FlowJo, cytolytics, ImageJ, Qupath 0.5.

For manuscripts utilizing custom algorithms or software that are central to the research but not yet described in published literature, software must be made available to editors and reviewers. We strongly encourage code deposition in a community repository (e.g. GitHub). See the Nature Portfolio guidelines for submitting code & software for further information.

Data

Policy information about availability of data

All manuscripts must include a data availability statement. This statement should provide the following information, where applicable:

- Accession codes, unique identifiers, or web links for publicly available datasets
- A description of any restrictions on data availability
- For clinical datasets or third party data, please ensure that the statement adheres to our policy

Details on data availability, including accession codes, restrictions, and compliance with Nature’s policy for third-party datasets, are fully described in the Data Availability Statement within the manuscript.

Research involving human participants, their data, or biological material

Policy information about studies with human participants or human data. See also policy information about sex, gender (identity/presentation), and sexual orientation and race, ethnicity and racism.

Reporting on sex and gender	All participants were asked about their sex and gender during enrollment. However, to protect participant privacy and prevent potential re-identification, the corresponding variables (sex, gender, and age) were not included in Supplementary Data 1.
Reporting on race, ethnicity, or other socially relevant groupings	95% of FAP participants and 100% of non-FAP participants self-identified as Caucasian (Supplementary Data 1). Therefore, no additional confounding factors related to race or ethnicity were considered in this study.
Population characteristics	All FAP participants had a clinically confirmed diagnosis of familial adenomatous polyposis, and non-FAP controls were free of hereditary colorectal tumor syndromes. Duodenal samples included macroscopically normal tissue from FAP patients (n=101; 49 female; mean age 39.4 [16–81]) and non-FAP controls (n=42; 25 female; mean age 47.4 [15–75]), duodenal adenomas from FAP patients (n=32; 18 female; mean age 40.5 [17–73]), and one FAP patient (female, age 67) with duodenal carcinoma. Colonic samples comprised normal tissue from FAP patients (n=24; 13 female; mean age 27.8 [16–69]) and non-FAP controls (n=10; 7 female; mean age 40.4 [23–62]), as well as colonic adenomas from FAP patients (n=13; 7 female; mean age 23 [20–39]). To comply with patient consent restrictions and reduce identification risks, age and gender/sex were omitted from Supplementary Data 1, and our analysis confirmed no association between these factors and key study parameters, as presented during review.
Recruitment	Participants were recruited from one of Germany's few specialized hereditary tumor syndrome outpatient units at the University Hospital Bonn, which treats the majority of patients with these rare conditions nationwide. Because all eligible individuals were systematically approached, the likelihood of selection bias is minimized.
Ethics oversight	The study was approved by the Institutional Review Board of University of Bonn ethics committee , with ethics approval number [#079/13, #040/16, #275/13 and #493/20]. Written informed consent was obtained from all participants prior to sample collection.

Note that full information on the approval of the study protocol must also be provided in the manuscript.

Field-specific reporting

Please select the one below that is the best fit for your research. If you are not sure, read the appropriate sections before making your selection.

☒ Life sciences ☐ Behavioural & social sciences ☐ Ecological, evolutionary & environmental sciences

For a reference copy of the document with all sections, see nature.com/documents/nr-reporting-summary-flat.pdf

Life sciences study design

All studies must disclose on these points even when the disclosure is negative.

Sample size	The sample sizes were determined by the rarity of the disorder and the limited availability of suitable specimens that met stringent clinical and molecular criteria. In order to ensure maximal representation of the patient population, an exhaustive inclusion strategy was employed, incorporating all accessible, clinically annotated samples.While formal statistical power calculations were precluded due to the scarcity of cases, this approach aligns with established methodologies for rare disease research and ensures that the cohort captures the full phenotypic heterogeneity of the disorder. In order to mitigate potential bias and enhance the validity of comparative analyses, rigorous quality control measures were implemented, including stratification by key clinical variables (e.g. age, disease severity). This strategy achieves a balance between ethical constraints (avoiding redundant sampling) and scientific rigour, in accordance with guidelines for studies involving rare conditions.
Data exclusions	No data were excluded post-analysis. All specimens underwent pre-established quality control (e.g.,cell count, sequencing depth, RNA integrity). Retained samples met these thresholds, ensuring robustness and reflecting the disorder's heterogeneity, consistent with human study research standards.
Replication	All experiments were replicated independently across ≥2 batches, with study groups processed concurrently to minimize variability. Key findings were consistently reproduced using standardized protocols, and no discrepancies arose, confirming robust reproducibility under controlled conditions.
Randomization	Participants were allocated into predefined groups (FAP vs. non-FAP) based on clinical diagnosis, as randomization is inherently incompatible with observational study designs. A consecutive sampling strategy was used to minimize selection bias, with eligibility restricted to individuals meeting strict inclusion criteria (no prior duodenal surgery, no active trial participation). Covariates (e.g., age, sex) were subsequently accounted for in the analyses, ensuring that no systematic factors other than FAP status influenced group allocation.
Blinding	Blinding was precluded by the observational design (FAP/non-FAP groups defined clinically). To mitigate bias, samples were processed in randomized batches by personnel blinded to group labels during data collection. Anonymized datasets were analyzed independently, ensuring objectivity until final unblinding.

Reporting for specific materials, systems and methods

We require information from authors about some types of materials, experimental systems and methods used in many studies. Here, indicate whether each material, system or method listed is relevant to your study. If you are not sure if a list item applies to your research, read the appropriate section before selecting a response.

Materials & experimental systems

n/a	Involved in the study
☐	☒ Antibodies
☐	☒ Eukaryotic cell lines
☒	☐ Palaeontology and archaeology
☒	☐ Animals and other organisms
☐	☒ Clinical data
☒	☐ Dual use research of concern
☒	☐ Plants

Methods

n/a	Involved in the study
☒	☐ ChIP-seq
☐	☒ Flow cytometry
☒	☐ MRI-based neuroimaging

Antibodies

Antibodies used	c-MAF EF660 Invitrogen 2480320, CD103 AF-700 Novus Biologicals NBP1-97564AF700, CD117 (ckit) PE-Cy7 BioLegend® 313212, CD117 (ckit) PE-Vio615 Miltenyi Biotec 130-111-598, CD123 FITC BioLegend® 306014, CD127 BV605 BioLegend® 351334, CD127 Spark YG™ 581 BioLegend® 351368, CD14 FITC BioLegend® 301804, CD161 APC-Cy7 BioLegend® 339928, CD19 FITC BioLegend® 302206, CD196 (CCR6) PE/Fire640 BioLegend® 353449, CD199 (CCR9) APC/F750 BioLegend® 358928, CD1a FITC BioLegend® 300104, CD20 FITC Biolegend® 302304, CD200R1 BV421 Biolegend® 329314, CD294 (CRTH2) BV421 BD Bioscience 562992, CD294 (CRTH2) BV510 BioLegend® 350120, CD294 (CRTH2) BV711 BioLegend® 350124, CD294 (CRTH2) PerCP-Cy5.5 BioLegend® 350116, CD3 FITC BioLegend® 300406, CD303 (BDCA-2) FITC Miltenyi Biotec 130-090-510, CD303 (NKp44) APC BioLegend® 325109, CD336 (NKp44) BV605 BD Bioscience 744301, CD336 (NKp44) BV786 BD Bioscience 744304, CD336 (NKp44) PerCP-Cy5.5 BioLegend® 325114, CD34 FITC BioLegend® 343504, CD4 BUV805 BD Bioscience 612887, CD4 FITC BioLegend® 317408, CD4 PE-Cy7 BioLegend® 300512, CD45 BUV395 BD Bioscience 563792, CD45 BUV805 BD Bioscience 564914, CD49a PerCP-eFluor™710 Invitrogen 46-9490-42, CD5 FITC BioLegend® 300606, CD5 BUV805 BD Bioscience 748492, CD56 BUV563 BD Bioscience 565704(612928), CD66b FITC BioLegend® 305104, CD8a SparkBlue-550 BioLegend® 344760, CD94 BUV737 BD Bioscience 748787, CD94 FITC BD Bioscience 555888, CXCR3 BV605 BioLegend® 353727, CXCR6 BUV661 BD Bioscience 749948, Epcam (CD326) BV421 BioLegend® 324220, FcεR1α FITC BioLegend® 334608, GATA3 BUV395 BD Bioscience 565448, HLA-DR BV570 BioLegend® 307638, IFN-γ BV421 BioLegend® 502532, IL-17A PE BioLegend® 512306, IL-17A PerCP-Cy5.5 BioLegend® 512314, IL-2 BV650 BioLegend® 500334, IL-22 APC Invitrogen 17-7222-82, IL-8 PE-Cy7 BioLegend® 511416, IL1R1 PE R&D Systems® FAB269P-100, Integrin ß7 PE/D594 BioLegend® 321226, KI67 BV750 BioLegend® 350536, KLRG1 BV421 BioLegend® 367706, NKp46 Pac Blue BioLegend® 331911, NKp80 FITC Miltenyi Biotec 130-094-843, NKp80 PE Miltenyi Biotec 130-112-590, NKp80 PE-Vio770 Milteny Biotec 130-105-068, Rorgt(RORγt) BV421 BD Bioscience 563282, Tbet BV711 BD Bioscience 563320, TCRαβ FITC BioLegend® 306706, TCRγδ FITC BioLegend® 331208, TNFα BV785 BioLegend® 502948, CD117 PE Miltenyi Biotec 130-113-544, CD14 PE Miltenyi Biotec 130-113-147, CD16 PE Miltenyi Biotec 130-113-393, CD3 PE Miltenyi Biotec 130-113-139, CD31 PE Miltenyi Biotec 130-111-540, CD45 PE R and D Systems NBP2-34527PE, EpCAM PE Miltenyi Biotec 130-110-999, HLA-DR.DP.DQ PE Miltenyi Biotec 130-120-715, NKp44-PE PE Miltenyi Biotec 130-120-486, CD19 PE PE Miltenyi Biotec 170-081-066, Ki67-FITC FITC DAKO F726801-8, aSMA-FITC FITC Thermo Fisher 30026304, anti-Epcam uncoupled InvitrogenTM MA1-10196, anti-Lysozyme uncoupled NovusTMBiologicals NBP2-61118, anti-Muc2 uncoupled Thermo Fisher Scientific MA5-32654, anti-IL17A uncoupled R&D Systems® AF-317-SP, anti-IL17A uncoupled Abcam AB79056, anti-CD3 uncoupled Leica NCL-L-CD3-565, anti-Duox2 uncoupled NovusTMBiologicals NB110-61576, Phospho-Histone H2A.X (Ser139) uncoupled Invitrogen MA1-2022, anti-IL17F uncoupled R&D Systems® MAB13352
Validation	Antibodies validation by manufacturer is presented in Supplementary Table 4

Eukaryotic cell lines

Policy information about cell lines and Sex and Gender in Research

Cell line source(s)	OP9-DL1 and OP9-DL4
Authentication	The OP9-DL1 and OP9-DL4 cell lines were generously provided by Prof. Dr. Juan Carlos Zúñiga-Pflücker (Sunnybrook Research Institute), Prof. Markus Uhrberg (University Hospital Düsseldorf), and Prof. Andreas Diefenbach (Charité – Universitätsmedizin Berlin), and were tested for Notch ligand expression (DLL1 & DLL4) by PCR prior to use.
Mycoplasma contamination	The cell lines were regularly tested for Mycoplasma contamination using a PCR kit.
Commonly misidentified lines (See ICLAC register)	The cell lines used in this study are not listed as commonly misidentified cell lines.

Clinical data

Policy information about clinical studies

All manuscripts should comply with the ICMJEguidelines for publication of clinical research and a completedCONSORT checklist must be included with all submissions.

Clinical trial registration	*Provide the trial registration number from ClinicalTrials.gov or an equivalent agency.*
Study protocol	*Note where the full trial protocol can be accessed OR if not available, explain why.*
Data collection	*Describe the settings and locales of data collection, noting the time periods of recruitment and data collection.*
Outcomes	*Describe how you pre-defined primary and secondary outcome measures and how you assessed these measures.*

Plants

Seed stocks	*Report on the source of all seed stocks or other plant material used. If applicable, state the seed stock centre and catalogue number. If plant specimens were collected from the field, describe the collection location, date and sampling procedures.*
Novel plant genotypes	*Describe the methods by which all novel plant genotypes were produced. This includes those generated by transgenic approaches, gene editing, chemical/radiation-based mutagenesis and hybridization. For transgenic lines, describe the transformation method, the number of independent lines analyzed and the generation upon which experiments were performed. For gene-edited lines, describe the editor used, the endogenous sequence targeted for editing, the targeting guide RNA sequence (if applicable) and how the editor was applied.*
Authentication	*Describe any authentication procedures for each seed stock used or novel genotype generated. Describe any experiments used to assess the effect of a mutation and, where applicable, how potential secondary effects (e.g. second site T-DNA insertions, mosiacism, off-target gene editing) were examined.*

Flow Cytometry

Plots

Confirm that:

- [x] The axis labels state the marker and fluorochrome used (e.g. CD4-FITC).
- [x] The axis scales are clearly visible. Include numbers along axes only for bottom left plot of group (a 'group' is an analysis of identical markers).
- [x] All plots are contour plots with outliers or pseudocolor plots.
- [x] A numerical value for number of cells or percentage (with statistics) is provided.

Methodology

Sample preparation	The biological source of of the cells and the tissue processing steps are included in in the Methods section .
Instrument	BD Fortessa Analyze, Sony ID700o 7L and BD FACSAria Fusion Sorter .
Software	Microsoft Office<br>GraphPad Prism<br>FlowJo<br>cytolytics
Cell population abundance	We routinely achieved >98% purity of of sorted populations.
Gating strategy	The gating information is is provided in in Fig.1 and Supplementary Fig.9.

- [x] Tick this box to confirm that a figure exemplifying the gating strategy is provided in the Supplementary Information.
